# Workplace violence in primary hospitals and associated risk factors: A cross‐sectional study

**DOI:** 10.1002/nop2.1090

**Published:** 2021-10-16

**Authors:** Hongfang Zhu, Xiaona Liu, Linyan Yao, Liping Zhou, Jianfen Qin, Chenping Zhu, Zhihong Ye, Hongying Pan

**Affiliations:** ^1^ Sir Run Run Shaw Hospital School of Medicine Zhejiang University Hangzhou China

**Keywords:** cross‐sectional study, medical staff, nurses, primary hospital, workplace violence

## Abstract

**Aim:**

To investigate the characteristics of workplace violence at primary hospitals in Southeast China and identify associated risk factors.

**Design:**

A cross‐sectional survey design was used for this work.

**Methods:**

We distributed a workplace violence questionnaire among medical staff at primary hospitals in Southeast Zhejiang Province, China. The data were collected between December 2016 and December 2017. We analysed the categorical data by using the chi‐square test and expressed it as frequencies. The risk factors were analysed by using multiple logistic regression analysis.

**Results:**

Among the 2,560 questionnaires, 1,842 (71.9%) medical staff indicated that they had experienced workplace violence. Verbal assault was the most common type, followed by physical and sexual assault. Furthermore, gender, age, marital status, education, technical position and number of hospital beds' numbers were independent risk factors.

## 1 INTRODUCTION

Workplace violence (WPV) in hospitals has become a global problem (Spector et al., 2014), but the incidence thereof in Chinese hospitals is much higher than in other parts of the world (Arnetz et al., 2015). The World Health Organization has defined that hospital WPV as the action that takes place when “*a medical practitioner* is insulted, threatened and attacked in the workplace poses a challenge for his safety, well‐being and health” (Bowers et al., 2007). This type of violence can be physical, sexual or psychological (Campbell et al., 2011).

With the rapid growth of China's social economy, the relationship between medical staff and patients has become increasingly tense, leading to a breakdown in trust crisis between them. However, Chinese studies have not been sufficiently focussed on the characteristics of violence, related risk factors (Liu et al., 2015) or systematic research in primary hospitals. Of all the medical settings, the primary ones are the most vulnerable in China. Therefore, to prompt primary hospitals to concentrate more on WPV prevention and control, we aimed to investigate the incidence of violence and associated risk factors among medical personnel in these hospitals in China. The following workplace violence inclusion criteria were established based on the World Health Organization definition of violence in the workplace: (a) psychological, physical and/or sexual violence suffered by hospital staff on hospital premises; (b) the violence occurred in the 12 months prior to the survey; (c) the victim must be a hospital staff member; (d) the place of violence is limited to hospitals; (e) the time limit is 12 months before the survey.

## 2 THE STUDY

### 2.1 Design and setting

This cross‐sectional study was conducted in four county‐level primary hospitals, namely the Jiangshan, Wuyi, Yongkang and Fuyang Hospitals in Zhejiang Province.

### 2.2 Participants

Based on the World Health Organization's (WHO) definition of WPV, we used the following inclusion criteria in our study: (a) hospital staff members who were victims of (b) psychological, physical and/or sexual violence, (c) on hospital premises, (d) in the 12 months prior to the survey. The data were collected from registered medical staff at county‐level primary hospitals in the Southeast Zhejiang Province who had more than 1 year of work experience.

### 2.3 Measures: Hospital Workplace Violence Questionnaire

The Hospital WPV Questionnaire was designed by Chen during his doctoral dissertation to study WPV among 7,198 medical workers in 20 different medical facilities. We obtained permission from the author and used the questionnaire to investigate the characteristics of, and staff responses to, WPV in the hospitals. The questionnaire includes two sections with 38 items. Part One (nine items) reflects the different types of WPV with two psychological violence (insults and threats); four physical violence (physical attacks without injuries; physical attacks with mild, visible or serious injuries); and three for sexual violence (sexual harassment or flirtation; assault; and attempted rape or rape) items. Part Two (14 items) mainly describes the victims' most severe WPV incident in the 12 months preceding the survey. Part Three (15 items) is about participants' personal data, their WPV knowledge and the hospital's response. The retest reliability of the questionnaire was 0.803. Results showed that the validity and reliability of the questionnaire were good, and we obtained consent from the original author to use the questionnaire.

### 2.4 Ethical considerations

The Ethics Committee of Sir Run Run Shaw Hospital, School of Medicine, Zhejiang University (20171120‐8) approved the study. Before we distributed the questionnaires, we assured the medical staff that the questionnaire will be used for academic research, their personal information will remain confidential, and they could withdraw at any stage. Moreover, the participants signed informed consent forms.

### 2.5 Data collection

We collected the data between December 2016 and 2017 by distributing 2,700 questionnaires of which 2,560 (94.8%) valid ones were returned (Table 1). The questionnaires were anonymized and sealed and posited by the participants into a specific box at a nursing station within 2 weeks of receiving the questionnaire.

**TABLE 1:** Demographic information (*N* = 2,560)

	Number	Rate
Gender		
Male	557	21.7%
Female	2,003	78.2%
Age		
<25	568	22.1%
25–30	900	35.1%
31–35	488	19.0%
36–40	234	9.1%
41–45	218	8.5%
46–50	86	3.3%
51–55	46	1.7%
56–60	9	0.3%
>60	11	0.4%
Marriage		
Unmarried	946	36.9%
Married	1,579	61.6%
Divorce or bereavement	35	1.3%
Education		
Technical secondary school	395	15.4%
Junior college	956	37.3%
Bachelor degree	1,168	45.6%
Master's degree	39	1.5%
PHD	2	<0.1%
Job		
Doctor	904	35.3%
Nurse	1,555	60.7%
Nurse assistant	8	0.3%
Medical laboratory	93	3.6%
Working year		
<1	263	10.2%
1–2	564	22.0%
3–5	377	14.7%
6–10	565	22.0%
11–15	320	12.5%
16–20	210	8.2%
21–25	127	4.9%
>25	134	5.2%
Technical post		
Undefined	336	13.1%
Junior	1,411	55.1%
Medium	577	22.5%
Senior‐deputy	196	7.6%
Senior	40	1.5%
Employ		
Regular	1,566	61.1%
Contract	825	32.2%
Temporary	101	3.9%
Others	68	2.6%
Hospital's beds number		
≤100	390	14.9%
101–300	92	3.5%
301–500	169	6.4%
501–700	1,041	39.9%
701–1,000	644	24.7%
>1,000	222	8.5%

### 2.6 Data analysis

Descriptive statistics were used to analyse the demographic data and WPV incidence. Moreover, we analysed the risk factors using multiple logistic regression analysis by applying SPSS version 21 (IBM) zero statistical software package (IBM Corporation), and *p* < .05 was considered statistically significant.

## 3 RESULTS

### 3.1 Sociodemographic data

Most of the participants were women (*N* = 2,003; 78.2%), married (*N* = 1,579; 61.6%) and nurses (*N* = 1,555; 60.7%). Almost half of them (45. 6%) held a bachelor's degree. Table 1 presents the biographic details of the participants. (*p* < .001).

### 3.2 WPV‐type incidence

Many participants (*N* = 1,842; 71.9%) experienced violence in previous year. The most common form of verbal assault was cursing (48.6%). Concerning physical violence, 156 participants were subjected to it without sustaining physical injuries. Physical violence that resulted in severe physical disability occurred in 23 participants. Notably, sexual violence was the least frequent. Results indicated only one sexual harassment incidence, with the highest incidence of 1.3%. Moreover, 15 (0.8%) of the medical staff had been subjected to more than one rape (or attempted rape). See Table 2.

**TABLE 2:** Types and incidence of WPV (*N* = 3,783)

Violence style	Sort	Frequency/year (%)				
		Once	2–3	4–5	6–10	>10
Verbal assault	Curse	448 (11.8)	664 (17.6)	284 (7.5)	119 (3.1)	327 (8.6)
	Threaten	569 (15.0)	495 (13.1)	152 (4.0)	66 (1.7)	126 (3.3)
Physical assault	No injury	156 (4.1)	72 (1.9)	15 (0.4)	4 (0.1)	4 (0.1)
	Mild injury	77 (2.0)	31 (0.8)	5 (0.1)	1 (~)	3 (~)
	Moderate injury	23 (0.6)	4 (0.1)	2 (~)	0	1 (~)
	Severe injury	17 (0.4)	4 (0.1)	1 (~)	0	1 (~)
Sex assault	Sex harassment	49 (1.3)	19 (0.5)	3 (0.1)	1 (~)	1 (~)
	Sex raid	16 (0.4)	5 (0.1)	2 (~)	0	1 (~)
	Rape or attempted rape	12 (0.3)	3 (0.1)	0	0	0

Note

“~”means the number is less than 0.1%.

Abbreviation: WPV, Workplace violence.

### 3.3 WPV‐related risk factors

When we compared the characteristics of the patients who had experienced WPV with those who had not, we found significant differences in gender, age, marriage, education, technical position and number of hospital beds (*p* < .05). However, no significant differences were observed in terms of job type, employment patterns and years of work experience (Table 3). Multiple logistic regression analysis revealed the following independent risk factors for WPV: gender (odds ratio [OR], 0.060; 95% confidence interval [CI], 0.037–0.0961; *p* < .001), age (OR, 68.026;95% CI, 43.961–105.266; *p* < .001), marital status (OR, 0.302; 95% CI, 0.230–0.397; *p* < .001), education (OR, 0.768; 95%CI, 0.686–0.860; *p* < .001), technical position (OR, 0.569; 95% CI, 0.453–0.714; *p* < .001) and the number of hospital beds (OR, 1.126; 95%CI, 1.037–1.221; *p* = .005) were identified as independent risk factors for WPV. See (Table 4).

**TABLE 3:** Univariate analysis of WPV in primary hospitals

Parameter	Non‐WPV	WPV	x^2^/t/z	*p*
Gender				
Male	156	401	24.525	<.001
Female	562	1,441		
Age				
<25	203	365	4.108	<.001
25–30	233	667		
31–35	140	348		
36–40	53	181		
41–45	46	172		
46–50	17	69		
51–55	10	36		
56–60	5	4		
>60	11	0		
Marriage				
Unmarried	297	649	21.711	<.001
Married	405	1,174		
Divorce or bereavement	16	19		
Education				
Technical secondary school	128	267	4.732	.001
Junior college	282	674		
Bachelor degree	303	865		
Master's degree	5	34		
PHD	0	2		
Job				
Doctor	238	666	0.892	.410
Nurse	435	1,120		
Nurse assistant	1	7		
Medical laboratory	44	49		
Working year				
<1	100	163	1.232	.283
1–2	165	399		
3–5	112	265		
6–10	154	411		
11–15	78	242		
16–20	43	167		
21–25	26	101		
>25	40	94		
Technical post				
Undefined	124	212	2.732	.043
Junior	414	997		
Medium	151	426		
Senior‐deputy	26	170		
Senior	3	37		
Employ patterns				
Formal	481	1,085	3.772	.337
Contract	179	646		
Temporary	41	60		
Others	17	51		
Hospital's beds number				
≤100	164	226	4.383	<.001
101–300	53	39		
301–500	33	136		
501–700	208	833		
701–1,000	186	458		
>1,000	74	148		

Abbreviation: WPV, Workplace violence.

**TABLE 4:** Multiple analysis of WPV in primary hospitals

Parameter	B	SE	Wald x^2^	*p*	OR	95%CI Upper	Lower
Gender	−2.813	.240	137.292	<.001	0.060	0.096	0.037
Age	4.220	.223	358.880	<.001	68.026	105.266	43.961
Marriage	−1.198	.139	73.737	<.001	0.302	0.397	0.230
Education	−0.264	.057	21.114	<.001	0.768	0.860	0.686
Technical post	−0.564	.116	23.719	<.001	0.453	0.714	0.453
Hospital's beds number	0.118	.042	8.068	.005	1.037	1.221	1.037

Abbreviations: CI, confidence interval; OR, odds ratio; WPV, Workplace violence.

## 4 DISCUSSION

Primary hospitals are particularly vulnerable settings for WPV, with a general WPV prevalence as high as 60%. Alarmingly, we found a 4% prevalence rate over a one‐year period. Furthermore, previous studies focussed on clinical settings in medium and large hospitals, but addressed limited types of WPV. That partly accounts for the relatively low prevalence of WPV. We investigated all common WPV styles and associated risk factors at several primary hospitals, which would account for a high event incidence.

Similar to previous studies (Yang et al., 2018) and consistent with regular conflicts in Chinese daily life, verbal assault was the most prevalent type of WPV, followed by physical and sexual assault. However, even though verbal assault is common in primary medical settings, psychological violence usually does not cause serious physical harm. Considerable domestic and foreign research reported that medical staff who experience prolonged verbal assault will experience low job satisfaction (Hanson et al., 2015), and professional fatigue (Heponiemi et al., 2014), or resort to resigning (Kim et al., 2018). Unfortunately, it is often not addressed and hospitals lack effective treatments.

Concerning physical violence, mild physical assault was mostly caused by pushing and shoving, which culminated in pain, bruises, scrapes or blemishes. However, moderate physical assault was often related to injuries such as wounds, fractures and internal organ or head injuries. Furthermore, severe physical assault was mostly caused by violent physical fighting which is harmful and holds serious consequences, including dysfunction or permanent disability. Although no fatal WPV cases were found, 23 of the medical staff had suffered from severe physical violence. Notably, these results only refer to the 12‐month period preceding the survey.

Sexual violence is often a "blind spot" in research. Many women do not report such incidences due to shame, or fear of being condemned or not believed (Arnetz & Arnetz, 2001). Furthermore, researchers from different cultural backgrounds provide different definitions of sexual violence. Chinese research reports on sexual violence might not be presenting the full picture. Concerningly, our findings showed that fifteen staff members had experienced rape (or attempted rape) in the preceding 12 months. However, due to the anonymity of this investigation, no follow‐up can be conducted for such vicious violence incidents.

Research has shown that women are more vulnerable to attacks (Guay et al., 2016; Lipscomb et al., 2006)—most registered nurses in China are female. However, in our study, not only gender but also age, marital status, education, position and the number of hospital beds were significantly correlated with the WPV incidence in primary hospitals.

In terms of age, younger, inexperienced medical staff are not well versed in communicating with problematic patients, which results in medical disputes. The disputes seem to evolve around errors that have occurred (Shi et al., 2015) probably due to limited work experience and sense of responsibility. Notably, senior and experienced medical staff are more likely to gain patients' trust than novice ones. This result is consistent with other research (Svoboda, 2013).

Regular workers are a high‐risk WPV group. This is related to the fact that they undertake clinical work and face patients for a prolonged time. Because of this, the doctors and nurses who have most access to patients are most vulnerable to WPV (Bruns et al., 2007; Stevenson et al., 2015). In Western countries, the contact between medical staff and patients centres around employment. Therefore, it is not often that medical staff tend to the same patients permanently. Technical positions are linked to education and working experience. Persons in more senior positions usually have advanced education and work experience, which enables them to deal better with high‐risk WPV patients.

Comprehensive measures are required to assist victims of violence. The first is soft support: this requires hospitals to regularly conduct training, set up professional courses and comprehensive emergency plans. The latter are led by the Security Department who conducts regular defence training and emergency drills for major violent incidents, to improve awareness.

The second is structural and systems support. Hospitals should establish a corresponding body to address the violence including a security alliance, consisting of nursing, medical affairs and security departments, to ensure that violent incidents are resolved the first time. For key departments with a high incidence of violence, a 24‐hr security guard system can be implemented, and monitoring equipment installed to ensure the safety of medical workers.

Third, psychological support should be made available to victims of violent incidences by a professional counselling team to provide trauma relief, reduce job burnout and emotional after‐effects. In addition, the hospital can provide facilities and equipment, such as boxing walls, or massage chairs, that is, a quiet and private environment where employees can vent their emotions, relax and reduce their stress. Appropriate holidays and subsidies are also advised. We hope that cooperation between parties can jointly create a violence‐free medical environment.

This study used the Hospital Workplace Violence Scale to investigate the prevention and response to violence in primary hospitals. It analyses the characteristics of violence, helps hospitals and medical administrative departments to formulate violence prevention and response training measures, and builds a violence prevention and response training system in grassroots hospitals. In the later stage, it can be used to establish a norm for the prevention and response of violence among grassroots medical staff in Zhejiang Province, providing a new theoretical basis for reducing the occurrence of violence and for the formulation of institutional policies by health administrative departments.

### 4.1 Limitations

As this exploratory study involved convenience sampling and was conducted at selected primary hospitals in the Zhejiang Province, central China, the sample population may not be representative of the Chinese population. Future research should involve other primary hospitals in China with a larger sample size and more rigorous questionnaire design. Moreover, due to the anonymity of this investigation, no follow‐up or support can be provided to the victims. However, follow‐up studies can be conducted for severe physical assault and rape incidences.

## 5 CONCLUSSION

The incidence of WPV was high in the primary hospitals in Zhejiang Province. Verbal assault was the most common type of WPV. To reduce or prevent the occurrence of WPV in primary hospitals, enhanced care should be provided to medical staff who are at risk, including females, younger, junior and medical staff. More studies are needed to ascertain the WPV risk factors in primary hospitals.

## CONFLICT OF INTEREST

The authors have no conflict of interest to declare.

## AUTHOR CONTRIBUTIONS

All authors have agreed on the final version and meet at least one of the following criteria (recommended by the ICMJE [http://www.icmje.org/recommendations/]): substantial contributions to conception and design, data acquisition or data analysis and interpretation; drafting or critically revising the article for important intellectual content.

## Data Availability

Some or all data, models or code generated or used during the study are available from the corresponding author by request.

## References

[nop21090-bib-0001] Arnetz, J. E. , & Arnetz, B. B. (2001). Violence towards health care staff and possible effects on the quality of patient care. Social Science and Medicine, 52(3), 417–427. 10.1016/S0277-9536(00)00146-5 11330776

[nop21090-bib-0002] Arnetz, J. E. , Hamblin, L. , Essenmacher, L. , Upfal, M. J. , Ager, J. , & Luborsky, M. (2015). Understanding patient‐to‐worker violence in hospitals: A qualitative analysis of documented incident reports. Journal of Advanced Nursing, 71(2), 338–348. 10.1111/jan.12494 25091833 PMC5006065

[nop21090-bib-0003] Bowers, L. , van der Werf, B. , Vokkolainen, A. , Muir‐Cochrane, E. , Allan, T. , & Alexander, J. (2007). International variation in containment measures for disturbed psychiatric inpatients: A comparative questionnaire survey. International Journal of Nursing Studies, 44(3), 357–364. 10.1016/j.ijnurstu.2006.01.005 16524581

[nop21090-bib-0004] Bruns, D. , Disorbio, J. M. , & Hanks, R. (2007). Chronic pain and violent ideation: Testing a model of patient violence. Pain Medicine, 8(3), 207–215. 10.1111/j.1526-4637.2006.00248.x 17371407

[nop21090-bib-0005] Campbell, J. C. , Messing, J. T. , Kub, J. , Agnew, J. , Fitzgerald, S. , Fowler, B. , Sheridan, D. , Lindauer, C. , Deaton, J. , & Bolyard, R. (2011). Workplace violence: Prevalence and risk factors in the safe at work study. Journal of Occupational and Environmental Medicine, 53(1), 82–89. 10.1097/JOM.0b013e3182028d55 21187791

[nop21090-bib-0006] Guay, S. , Goncalves, J. , & Boyer, R. (2016). Evaluation of an education and training program to prevent and manage patients' violence in a mental health setting: A pretest‐posttest intervention study. Healthcare, 4(3), 49. 10.3390/healthcare4030049 PMC5041050 27490582

[nop21090-bib-0007] Hanson, G. C. , Perrin, N. A. , Moss, H. , Laharnar, N. , & Glass, N. (2015). Workplace violence against homecare workers and its relationship with workers health outcomes: A cross‐sectional study. BMC Public Health, 15, 11. 25595487 10.1186/s12889-014-1340-7 PMC4308913

[nop21090-bib-0008] Heponiemi, T. , Kouvonen, A. , Virtanen, M. , Vanska, J. , & Elovainio, M. (2014). The prospective effects of workplace violence on physicians' job satisfaction and turnover intentions: the buffering effect of job control. BMC Health Services Research, 14, 19. 24438449 10.1186/1472-6963-14-19 PMC3898009

[nop21090-bib-0009] Kim, H. , Kim, J. S. , Choe, K. , Kwak, Y. , & Song, J. S. (2018). Mediating effects of workplace violence on the relationships between emotional labour and burnout among clinical nurses. Journal of Advanced Nursing, 74(10), 2331–2339. 10.1111/jan.13731 29869815

[nop21090-bib-0010] Lipscomb, J. , McPhaul, K. , Rosen, J. , Brown, J. G. , Choi, M. , Soeken, K. , Vignola, V. , Wagoner, D. , Foley, J. , & Porter, P. (2006). Violence prevention in the mental health setting: The New York state experience. Canadian Journal of Nursing Research, 38(4), 96–117. 17290957

[nop21090-bib-0011] Liu, H. , Zhao, S. , Jiao, M. , Wang, J. , Peters, D. H. , Qiao, H. , Zhao, Y. , Li, Y. , Song, L. , Xing, K. , Lu, Y. , & Wu, Q. (2015). Extent, nature, and risk factors of workplace violence in public tertiary hospitals in China: A cross‐sectional survey. International Journal of Environmental Research and Public Health, 12(6), 6801–6817. 10.3390/ijerph120606801 26086703 PMC4483731

[nop21090-bib-0012] Shi, J. , Wang, S. , Zhou, P. , Shi, L. , Zhang, Y. , Bai, F. , Xue, D. , & Zhang, X. (2015). The frequency of patient‐initiated violence and its psychological impact on physicians in china: A cross‐sectional study. PLoS One, 10(6), e0128394. 10.1371/journal.pone.0128394 26030143 PMC4450867

[nop21090-bib-0013] Spector, P. E. , Zhou, Z. E. , & Che, X. X. (2014). Nurse exposure to physical and nonphysical violence, bullying, and sexual harassment: A quantitative review. International Journal of Nursing Studies, 51(1), 72–84. 10.1016/j.ijnurstu.2013.01.010 23433725

[nop21090-bib-0014] Stevenson, K. N. , Jack, S. M. , O'Mara, L. , & LeGris, J. (2015). Registered nurses' experiences of patient violence on acute care psychiatric inpatient units: An interpretive descriptive study. BMC Nursing, 14(1), 10.1186/s12912-015-0079-5 PMC4440495 25999795

[nop21090-bib-0015] Svoboda, J. S. (2013). Circumcision of male infants as a human rights violation. Journal of Medical Ethics, 39(7), 469–474. 10.1136/medethics-2012-101229 23698885

[nop21090-bib-0016] Yang, B. X. , Stone, T. E. , Petrini, M. A. , & Morris, D. L. (2018). Incidence, type, related factors, and effect of workplace violence on mental health nurses: A cross‐sectional survey. Archives of Psychiatric Nursing, 32(1), 31–38. 10.1016/j.apnu.2017.09.013 29413069

